# Transcriptome Analysis of Microglia Reveals That the TLR2/IRF7 Signaling Axis Mediates Neuroinflammation After Subarachnoid Hemorrhage

**DOI:** 10.3389/fnagi.2021.645649

**Published:** 2021-06-30

**Authors:** Shenbin Xu, Shuhao Mei, Jianan Lu, Haijian Wu, Xiao Dong, Ligen Shi, Jingyi Zhou, Jianmin Zhang

**Affiliations:** ^1^Department of Neurosurgery, Second Affiliated Hospital, School of Medicine, Zhejiang University, Hangzhou, China; ^2^Brain Research Institute, Zhejiang University, Hangzhou, China; ^3^Collaborative Innovation Center for Brain Science, Zhejiang University, Hangzhou, China

**Keywords:** subarachnoid hemorrhage, microglia, flow cytometry, bulk RNA-seq, early brain injury

## Abstract

Microglia-mediated neuroinflammatory response in the early brain injury after subarachnoid hemorrhage (SAH) has been reported to have an impact on progress, and the mechanism is not completely understood. Here, we performed genome-wide transcriptome analysis of microglia purified from damaged hemisphere of adult mice at 3 days after SAH or sham operation. Robust transcriptional changes were observed between SAH-induced and healthy microglia, indicating rapid activation of microglia after suffering from SAH. We identified 1576 differentially expressed genes (DEGs; 928 upregulated and 648 downregulated) in SAH-induced microglia compared with sham microglia, representing a strong alteration of the genome (6.85% of total ∼23,000 genes). Functional enrichment of these DEGs indicated that cell division, inflammatory response, cytokine production, and leukocyte chemotaxis were strongly activated in SAH-induced microglia. Moreover, we identified and proved that the TLR2/IRF7 signaling axis was involved in the regulation of this microglia-mediated inflammation in SAH mice by performing flow cytometry and immunofluorescence. Together, these results provided a perspective of microglia-mediated neuroinflammatory response in the early stage of SAH and might give a new therapeutic target for SAH.

## Introduction

Subarachnoid hemorrhage (SAH), which is mainly caused by intracranial aneurysm rupture, is a severe subtype of stroke with high mortality, disability, and poor outcomes (Macdonald and Schweizer, 2017). It accounts for 5% of strokes and has an annual incidence of 6–10 cases per 100,000 persons (Amodio et al., 2020; Macdonald and Schweizer, 2017). Early brain injury (EBI), which occurs in the first 72 h after bleeding, has been considered as the most important pathophysiological mechanism contributing to delayed cerebral ischemia and poor prognosis (Rass and Helbok, 2019). EBI is associated with many pathological processes, such as neuroinflammation, brain edema, global ischemia, and excitotoxicity (Fujii et al., 2013). Among them, neuroinflammation are considered to play a crucial role in EBI (Fujii et al., 2013). Microglia, the main resident immune cells in the central nervous system (CNS), is the most significant mediator in neuroinflammation. They constantly surveil the microenvironment and respond to damage and pathogens, acting as double-edged swords in different pathological states (Soulet and Rivest, 2008; Liu et al., 2019).

In the context of SAH, remarkable accumulation of microglia was observed within the first 3 days (Zheng et al., 2020) and lasted 28 days after bleeding (Schneider et al., 2015). In the acute phase, microglia are activated and secrete cytokines [e.g., interleukin-1β (IL-1β), IL-6, and tumor necrosis factor-α (TNF-α)], chemokines, and other potentially toxic chemicals, leading to inflammatory response and cell death (Schneider et al., 2015). However, microglia are also responsible for phagocytosis and clearance of blood and cell debris, indicating that microglia exhibit neuroprotective functions after bleeding (Schallner et al., 2015). Given the complex function of microglia, the specific role and underlying mechanisms of microglia in SAH remain largely obscure.

Toll-like receptors (TLRs), a class of pattern recognition receptors (PRRs) that are highly enriched in microglia, recognize the pathogen-associated molecular pattern (PAMP) ligands and the endogenous danger-associated molecular pattern (DAMP) ligands. Under pathological conditions, microglia initiate innate immune response *via* TLRs (Lalancette-Hebert et al., 2017). A previous study has shown that soluble TLR2 is elevated in cerebrospinal fluid (CSF) in patients with SAH (Sokol et al., 2016). 6-MP and glycyrrhizin treatment can attenuate TLR2 expression and SAH-induced brain injury (Chang et al., 2014, 2015). Additionally, TLR2 stimulation increases the leukocytosis in the CSF and blood flowing through choroid plexus (Rayasam et al., 2020). Interferon regulatory factor 7 (IRF7) is a multifunctional transcription factor that can be activated by PRRs (Ning et al., 2011). TLR2 can activate IRF7 in inflammatory monocytes and bone marrow-derived macrophages (Barbalat et al., 2009; Dietrich et al., 2010). Moreover, IRF7 participates in the M1-like microglial polarization switch (Tanaka et al., 2015). In conclusion, the TLR2/IRF7 signaling pathway may have influence on neuroinflammation. However, its exact function in mediating microglia, under SAH condition, is largely unknown.

To understand the microglial transcriptional changes after SAH and its potential role in SAH, we performed genome-wide transcriptome analysis of microglia isolated from damaged hemispheres of adult mice 3 days post-SAH and sham operation. We explored the functional implications of microglia in response to SAH and found that the microglia involved in neuroinflammation may be regulated by the TLR2/IRF7 signaling pathway.

## Materials and Methods

### Animal

Eight- to 10-week-old male C57BL/6 mice (SLAC Laboratory Animal Co., Ltd., Shanghai, China) were housed in a temperature- and humidity-controlled room under a 12-h day/night cycle and had free access to food and water. All protocols were approved by the Institutional Ethics Committee of the Second Affiliated Hospital, Zhejiang University School of Medicine. The animal experiments were performed according to the National Institutes of Health’s Guide for the Care and the Use of Laboratory Animals and the ARRIVE (Animal Research: Reporting *in vivo* Experiments) guidelines.

### Microglia Depletion

As previously described, PLX3397 (Selleckchem, Houston, TX, United States) was formulated in AIN-76 A standard chow at a concentration of 290 ppm. Mice were fed with PLX3397 chow for 21 consecutive days (Najafi et al., 2018) until the end of experiments.

### SAH Model

The endovascular perforation model was established as previously described (Muroi et al., 2015). Briefly, mice were anesthetized with pentobarbital sodium (40 mg/kg) *via* intraperitoneal injection. Left carotid artery and its bifurcation were exposed. Then, 5-0 sharpened nylon suture was inserted into the internal carotid artery (ICA) from the external carotid artery. Then, the suture was pushed until the tip reached the intracranial bifurcation of anterior cerebral artery and middle cerebral artery. The suture was pushed 1 mm further to perforate the vessel. The mice in the sham group underwent the same procedures except perforation. Since all procedures were performed on the left side, we collected the left hemisphere for all downstream experiments. Additionally, we assessed the degree of SAH *via* the grading system as previously described (Sugawara et al., 2008).

### Neurological Assessment

Modified Garcia test (range, 0–18) was used to assess the short-term neurological performance, by evaluating spontaneous activity, climbing, forelimb stretching, spontaneous movements of all limbs, body proprioception, and response to vibrissae touch (Shi et al., 2018).

### Adhesive Removal Test

To access the motor coordination and sensory neglect after SAH, adhesive test was performed following previous studies (Bouet et al., 2009). Small adhesive tape strips (2 mm × 3 mm) were applied to mice forepaws. Contact time and removal time were recorded with a maximum observation time of 120 s.

### Fluorescence-Activated Cell Sorting and Flow Cytometry

Mice were anesthetized with 40 mg/kg of pentobarbital sodium and transcardially perfused with ice-cold PBS. Brain tissues were obtained, and cerebrum was dissected and separated into ipsilateral and contralateral hemispheres. Ipsilateral hemispheres were mechanically dissociated using a razor blade and placed in a 15-ml conical tube with digestion solution [0.6 mg/ml of collagenase D (Sigma)]. Then, the mixture were incubated for 30 min at 37°C. After that, a 70-μm strainer was used to generate a single-cell suspension (BD FALCON). Cells were isolated by centrifugation (30 min, 800 × *g* at 23°C) using 30–70% Percoll gradient solutions (GE Healthcare) (Agalave et al., 2020). Isolated cells were washed and resuspended in PBS with 0.01% bovine serum albumin (BSA) and then incubated with indicated anti-mouse antibodies for 30 min at 4°C [rat anti-mouse CD45 PerCP (BD Bioscience) and rat anti-mouse CD11b FITC (BD Bioscience)]. The population of microglia (CD45 positive and CD11b positive) was sorted.

In flow cytometry, rat anti-mouse Ly6G PE and rat anti-mouse Ly6C APC (BD Bioscience) antibodies were used and incubated with CD45 perCP and CD11b FITC.

### RNA Extraction and Sorted Microglia Sequencing

Total RNA from microglia sorted by FACS was isolated using TRIzol (Invitrogen, CA, United States) according to the manufacturer’s protocol. The total RNA quantity and purity were checked by an Agilent 2100 bioanalyzer. High-quality samples (RIN number > 6.8) were used for downstream sequencing. Sequence libraries were constructed according to the standard SMART-seq protocol, and paired-end sequencing was performed with Illumina Novaseq 6000 (LC Bio) following the vendor’s recommended protocol. Prior to assembly, low-quality reads that contain sequencing adaptors, sequencing primers, or low-quality nucleotides, were removed. The sequence quality was also checked with FastQC. HISAT was used to align and map reads to the UCSC^1^ GRCm38 mouse reference genome. The mapped reads of each sample were assembled using StringTie. Then, all transcriptomes from the samples were merged to reconstruct a comprehensive transcriptome using perl scripts. After the final transcriptome was generated, StringTie and edgeR were used to estimate the expression levels of all transcripts. StringTie was used to perform expression level for mRNAs by calculating Fragment per Kilobase of transcript per Million mapped reads (FPKM). All raw sequence data have been uploaded to GSE167957.

### RNA-Seq Data Analysis

The expression matrixes were counter-checked to determine if there were any systematic errors or batch effects. The sva R package was used for identifying, estimating, and removing batch effects (Leek et al., 2012). The differentially expressed genes (DEGs) were selected with fold change > 2 or fold change < −2 and with statistical significance (Benjamini–Hochberg adjusted *p*-value < 0.01) by DESeq2 package (Love et al., 2014). FPKM were used for gene expression and were log2(*x* + 1) transformed. Principal components analysis (PCA) was performed on normalized counts. R package pheatmap was used to generate heatmap.

Functional enrichment analysis was conducted using *Metascape*^2^ (Bhattacharya et al., 2018). All genes in mouse genome were used as background genes, and the default settings were used as enrichment criteria (minimum overlap = 3, *P*-value cutoff = 0.01, minimum enrichment score = 1.5). A gene ontology term was considered activated/increased with a *z*-score > 2 and a *p*-value < 0.01, and was predicted inhibited/decreased with a *z*-score < −2 and a *p*-value < 0.01.

Transcription factors (TFs) of selected DEGs were predicted using IRegulon plugin in Cytoscape (Janky et al., 2014). TFs were ranked by normalized enrichment score (NES), and NES > 3 was set as threshold.

The protein–protein interaction (PPI) network of selected DEGs was established using The Search Tool for the Retrieval of Interacting Genes (STRING^3^) (Szklarczyk et al., 2015) and then visualized in Cytoscape (Shannon et al., 2003). A combined score > 0.4 was set as a significant threshold. Furthermore, CytoHubba, a Cytoscape plugin, was used to explore hub genes in the constructed PPI network, and the top 10 genes were displayed based on degree method.

### Immunofluorescence Staining

Immunofluorescence staining was performed as described previously (Lu et al., 2019). In brief, mice were sacrificed and transcardially perfused with 0.9% NaCl, followed by 4% paraformaldehyde. Brain tissues were harvested and immersed in 4% paraformaldehyde for 24 h and then cryoprotected in 30% sucrose solution. Frozen serial coronal brain sections (9 μm) were prepared and fixed on slides. Slices were blocked with 5% donkey serum for 1 h and then incubated at 4°C overnight with primary antibodies, including goat anti-Iba1 antibody (1:500, Abcam, ab5076), rabbit anti-Tlr2 antibody (1:100, Abcam, ab209216), and mice anti-Irf7 antibody (1:200, Santa Cruz, sc-74471). After washing, the cryosections were incubated at 37°C for 1 h with the following secondary antibodies: donkey-anti-goat IgG(H + L) Alexa Fluor 594 (1:500, Thermo Fisher, A-11058), donkey-anti-rabbit IgG (H + L) Alexa Fluor 488 (1:500, Thermo Fisher, A-21206), and goat-anti-mouse IgG (H + L) Alexa Fluor 488 (1:500, Thermo Fisher, A-11001). Finally, the sections were observed and images were taken using an Olympus fluorescence microscope (Olympus Co., Japan).

### Statistical Analysis

RNA-seq data were analyzed as mentioned above. For other data, the statistical analyses were conducted using R software (version 3.6.3) and GraphPad Prism (version 8.0.2). Student’s *t*-test and the Kruskal–Wallis test were employed in the two-group comparisons. A two-tailed *P*-value of <0.05 was considered statistically significant without specific annotation.

## Results

### Microglia Lead to Poor Neurological Outcome in Acute Stage After SAH

**FIGURE 1:**
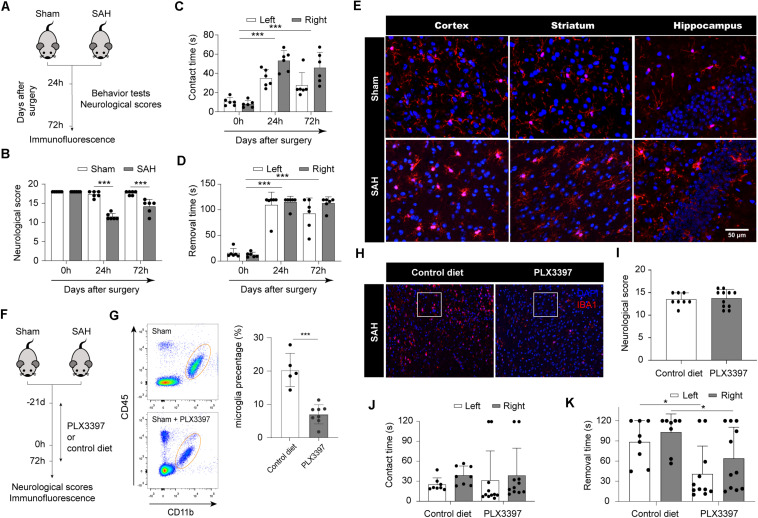
Microglia lead to poor neurological outcome in acute stage after SAH. **(A)** Schematic diagram of experimental design. **(B)** Comparison of neurologic deficit scores among sham and SAH groups at 1 and 3 days after SAH. **(C,D)** Comparison of adhesive removal test results among sham and SAH groups at 1 and 3 days after SAH. **(E)** Immunofluorescence staining results of microglia in sham and SAH mice at 3 days after SAH, *n* = 3 per mice group. **(F)** Schematic diagram of microglia clearance. **(G)** Flow cytometry results proved that PLX3397 can clear microglia cells. **(H)** Immunofluorescence staining results of microglia in SAH mice fed with PLX3397 or control diet at 3 days after SAH. **(I)** Comparison of neurologic deficit scores among control diet and PLX3397 groups at 3 days after SAH. **(J,K)** Comparison of adhesive removal test results among control diet and PLX3397 groups at 3 days after SAH. SAH, subarachnoid hemorrhage; *P < 0.05, ***P < 0.001.

At first, we induced SAH in adult C57BL/6 mice; the neurological scores and behavior tests were assessed in both SAH and sham group (Figure 1A). At 24 and 72 h after SAH, the average neurological scores were 11.714 ± 0.881 and 14.167 ± 1.675, respectively. SAH mice showed worse neurological scores than the sham group (*p* < 0.001; Figure 1B). At 24 h after SAH, compared with the sham group, SAH mice showed significantly increased contact times and removal times, respectively (*p* < 0.001; Figures 1C,D). Similarly, at 72 h, increased contact times and removal times were observed in SAH mice compared to sham mice (*p* < 0.001; Figures 1C,D). Representative images of Iba1 staining in cortex, striatum, and hippocampus are shown in Figure 1E. Significant morphological changes, like larger bodies, thicker pseudopodia, and ameboid morphology, were displayed in SAH mice, indicating microglia activation at 72 h after SAH.

To examine whether microglia play a detrimental role in SAH, we administrated the CSF1R inhibitor PLX3397 or control diet in chow to deplete microglia (Figure 1F). The number of microglia (CD45^*low*^/CD11b^+^) was assessed through flow cytometry. Administration of PLX3397 to sham mice resulted in an 65% reduction in microglia compared to control mice (*p* < 0.0001; Figure 1G). Next, we induced SAH in mice pretreated with PLX3397 or control diet, and maintained their chow until sacrifice (Figure 1F). Immunofluorescence showed that the number of microglia in the PLX3397-fed group was significantly lower than the control diet group at 72 h after SAH (Figure 1H). PLX3397 treatment had no apparent effect on the neurological scores at 72 h after SAH (Figure 1I). In the adhesive removal test, there was no difference in average contact times between the PLX3397-fed group and the control diet group (Figure 1J). However, PLX3397-fed mice spent less time removing the tape at 72 h after SAH compared with the control diet group (*p* < 0.05; Figure 1K). Taken together, these data suggested that microglia were activated after SAH, and they may positively correlate with neurological dysfunction.

### SAH Induces Strong Transcriptomic Alterations in Microglia

**FIGURE 2:**
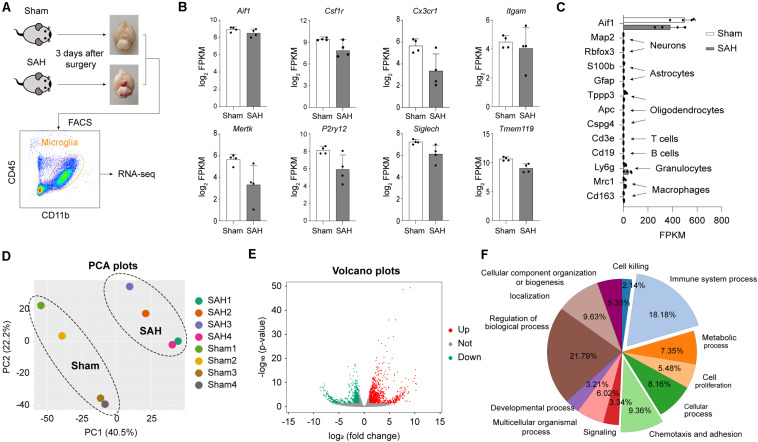
Subarachnoid hemorrhage induces strong transcriptomic alterations in microglia. **(A)** Microglia cell sorting strategy. **(B)** mRNA expression levels of microglia marker genes. **(C)** mRNA expression levels of microglia (*Aif1*), neuron (*Map2, Rabfox3*), astrocyte (*S100b*, *Gfap*), ependymal cell (*Tppp3*), oligodendrocyte progenitor cells (*Apc*, *Cspg4*), T cell (*Cd3e*), B cell (*Cd19*), neutrophil (*Ly6g*), macrophage (*Mrc1*, *Cd163*), and marker genes. **(D)** PCA plots of microglia transcriptome sequencing results. **(E)** Volcano plots show the DEGs (fold change > 2 or <–2, adj_pval < 0.01) in microglia from SAH brain versus sham brain. **(F)** Shown are the percentages of significantly overrepresented GO terms. Twelve major clusters of functions were identified.

To elucidate the functional roles of microglia after SAH, we used FACS to purify the microglia population (CD45^+^/CD11b^+^) from the brains of mice 72 h after SAH or sham operation. Sorted microglia cells were subjected to bulk RNA-seq (Figure 2A). We confirmed the high expression level of microglial marker in FACS-sorted cells, such as *Itgam* (encoding CD11b), *Cx3cr1*, *Aif1* (encoding Iba1), *Csf1r*, *Mertk*, *Tmem119*, *Siglech*, and *P2ry12* (Figure 2B). The established markers of neurons (*Map2* and *Rbfox3*), astrocytes (*S100b* and *Gfap*), oligodendrocytes (*Tppp3, Apc*, and *Cspg4*), and other immune cells (*Cd3e, Cd19, Ly6g, Mrc1*, and *Cd163*) were all expressed at low level (Figure 2C). The result of PCA performed on RNA-seq data showed that samples in the same group clustered together, and samples in different groups separated clearly (Figure 2D), indicating robust transcriptomic differences between the SAH and Sham group.

Next, to determine the transcriptomic changes of microglia induced by SAH, differential expression analysis was performed. A total of 1576 genes were identified as DEGs (fold change > 2 or <−2, Benjamini–Hochberg adjusted *p*-value < 0.01), which contained 928 upregulated genes and 648 downregulated genes in post-SAH microglia compared with sham hemispheres (Figure 2E and Supplementary Table 1), indicating strong alterations of the genome (6.85% of total ∼23,000 genes). To elucidate the functional alterations in post-SAH microglia, pathway enrichment analysis was performed on the DEGs by an online tool *Metascape*. Regulation of biological process (21.79%), immune system process (18.18%), and chemotaxis and adhesion (9.36%) accounted for a major proportion of GO terms obtained from *Metascape* (Figure 2F). The top 20 clustered GO terms and specific GO terms obtained by *Metascape* are shown in Supplementary Tables 2, 3, respectively.

### Immune Response and Chemotaxis Were Activated in Post-SAH Microglia

**FIGURE 3:**
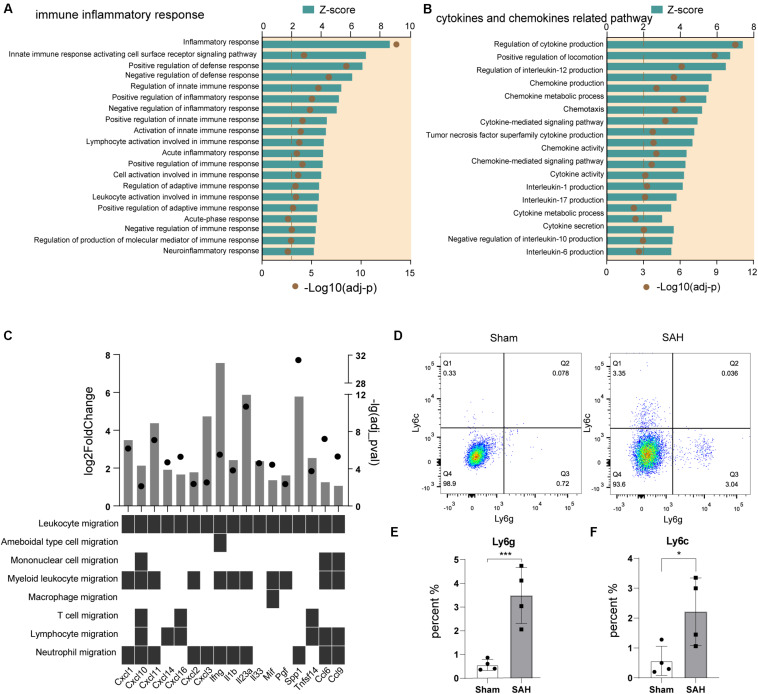
Immune response and chemotaxis term were activated in post-SAH microglia. **(A)** GO enrichment analysis was performed by Metascape on all DEGs in sham and SAH microglia. Shown are the *z*-scores of biological functions on inflammatory and immune response-related pathways. **(B)** Shown are the *z*-scores of biological functions on cytokine and chemokine-related pathways. **(C)** Expression profiles of DEGs (fold change > 2, adj_pval < 0.01) in SAH microglia related to leukocyte recruitment pathways. **(D–F)** Flow cytometric analysis showed that the number of Ly6c+ and Ly6g+ cells increased after SAH, *n* = 4 per mice group. DEG, differentially expressed gene. *P < 0.05, ***P < 0.001.

We analyzed the biological processes related to *immune inflammatory responses* in microglia after SAH, and the Top 20 GO terms predicted to be activated (*z*-score > 2) are shown in Figure 3A. The inflammatory response, defense response, innate immune response, adaptive immune response, and neuroinflammatory response were all involved.

As chemotaxis was a large cluster that participates in immune inflammatory response, we further compared the pathways that related to cytokines and chemokines. 17 GO terms were exhibited in Figure 3B, all of them were predicted to be activated (*z*-score > 2). We also screened the expression level of a panel of cytokine and chemokine genes (Bhattacharya et al., 2018), and there were 17 significantly upregulated genes. The upregulated genes involved seven chemokine-encoding genes (*Cxcl1*, *Cxcl2*, *Cxcl3*, *Cxcl10*, *Cxcl11*, *Cxcl14*, and *Cxcl16*), and 10 cytokine-encoding genes, such as *Ccl6*, *Ccl9*, *Il1b*, *Il23a*, *Ifng*, *Il33*, and *Mif* (Figure 3C). These data indicated that a variety of pro-inflammatory cytokines and chemokines were released from post-SAH microglia, to recruit peripheral immune cells into brain parenchyma, and it also suggested that myeloid cells were the main target of upregulated cytokines and chemokines (Figure 3C). Then, we detected the different populations of myeloid cells in ipsilateral hemispheres of SAH and sham mice by flow cytometry. Compared to sham mice, there were significant increases in the amount of infiltrating immune cells in post-SAH hemispheres, such as CD45^+^Ly6C^+^Ly6G^–^ monocytes (*p* < 0.01) and CD45^+^Ly6C^–^Ly6G^+^ neutrophils (*p* < 0.05) (Figures 3D–F).

### Irf7 Is a Master Regulator Involved in the Post-SAH Microglia

**FIGURE 4:**
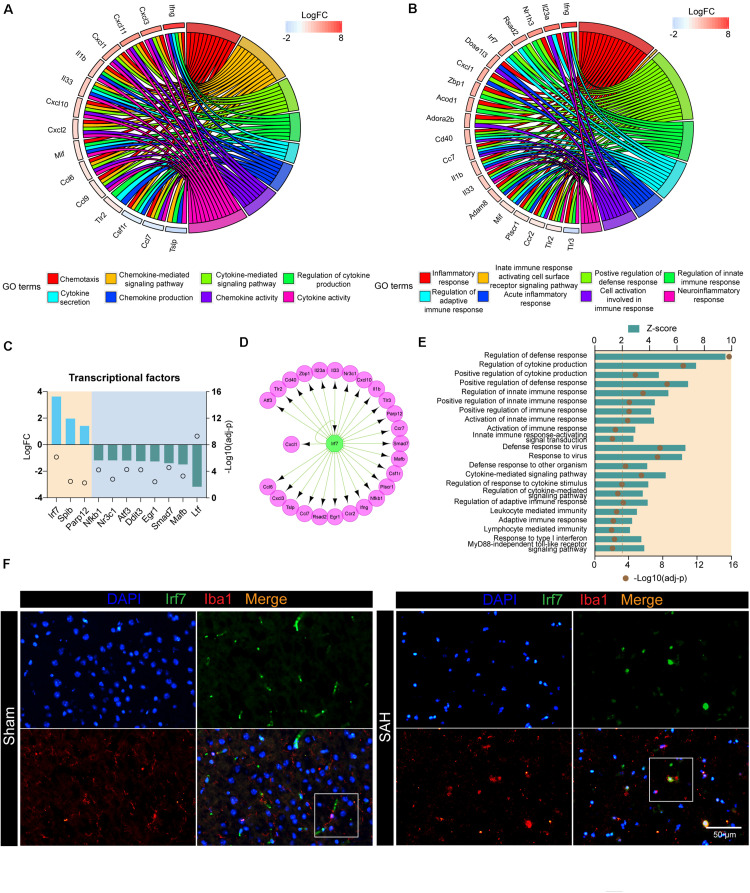
Irf7 is a transcription factor involved in the post-SAH microglia. **(A)** Circular plot showed the DEGs involved in cytokine- and chemokine-related pathways. **(B)** Circular plot showed the DEGs involved in inflammatory and immune response-related pathways. **(C)** Expression profiles of differentially expressed transcriptional factors (fold change > 2, adj_pval < 0.01) in SAH microglia. **(D)** Regulatory network of *Irf7*. **(E)** Shown are the *z*-scores of biological functions that are correlated with *Irf7*. **(F)** Immunofluorescence staining results of Irf7 and Iba1 in sham and ICH mice, *n* = 3 per mice group. DEG, differentially expressed gene.

Next, we investigated the DEGs involved in cytokine- and chemokine-related pathways. These upregulated products could also be defined as receptors (e.g., TLR2), cytokines (e.g., IFNG and IL1B), and chemokines (e.g., CXCL1 and CXCL10). The top 15 genes, including *Ifng*, *Cxcl1*, *Cxcl10*, *Tlr2*, and *Il1b*, were associated with at least three functional subcategories of cytokine- and chemokine-related pathways (Figure 4A). In addition, we also screened out the genes that contribute multiple steps in immune inflammatory response, including inflammatory response, regulation of innate immune response, neuroinflammatory response, and so on (Figure 4B). The products of these genes predicted to be active included TFs (e.g., NR1H3 and IRF7), receptors on the membrane (e.g., CD40 and TLR2), nuclear receptor (e.g., PLSCR1), enzyme (e.g., DNASE1L3), and inflammatory-related proteins (e.g., ADAM8, RSAD2, and ZBP1).

In order to investigate the upstream regulators of these biological processes, the interactions between all DEGs, which consisted of 985 nodes and 11,456 edges, were constructed based on STRING database (Supplementary Table 5). Then, the nodes related to high-frequency DEGs in Figures 4A,B were retrieved, out of which 11 unigenes were TFs (Figure 4C). In our dataset, the expression of *Irf7*, *Spib*, and *Parp12* were significantly upregulated, whereas *Nfkb1* was downregulated in post-SAH microglia. The upstream regulator analysis was performed by IRegulon, a plugin in Cytoscape. The top 10 potential TFs that target the selected DEGs are shown in Table 1. According to the transcriptional level and the results from IRegulon, IRF7 was predicted to be a potent master regulator of the transcriptional processes related to *immune inflammatory response* and *chemotaxis*. In the TF target gene network, IRF7 was predicted to target 19 genes in the selected high-frequency DEGs (e.g., *Cd40*, *Il1b*, *Cxcl10*, and *Il23a*) (Figure 4D). Moreover, Figure 4E demonstrated that IRF7 involved a GO term that was predicted to be activated. Using dual immunofluorescence staining of Iba1 and IRF7, we confirmed that IRF7 expression was induced in microglia at 72 h after SAH (Figure 4F). IRF7 immunosignal was detected in the cortex from sham mice, while the IRF7 was hardly colocalized with Iba1.

**TABLE 1:** Top 10 transcription factors obtained by IRegulon.

Transcription factor	NES	Target genes
*Irf8*	8.7502	*Parp12, Zbp1, Tlr3, Atf3, Cxcl10, Cd40, Ccr7, Irf7, Ccl6, Ccr2, Tlr2, Mafb, Egr1, Nr3c1, Il33, Tslp, Rsad2*
*Nfkb2*	8.20316	*Cxcl10, Egr1, Nfkb1, Cd40, Cxcl1, Il1b, Cxcl3, Tslp, Ccr7, Atf3, Cxcl2, Spib, Smad7, Plscr1, Il23a, Tlr2, Csf1r, Mafb, Il33*
*Irf7*	6, 986	*Atf3, Tlr2, Cd40, Zbp1, Il23a, Irf7, Il33, Nr3c1, Cxcl10, Il1b, Tlr3, Parp12, Ccr7, Smad7, Mafb, Csf1r, Plscr1, Nfkb1, Ifng, Ccr2, Egr1, Rsad2, Ccl6, Ccl7, Tslp, Cxcl3, Cxcl1*
*Homez*	4.708	*Nr3c1, Egr1, Smad7, Mafb, Atf3, Csf1r, Ccr7, Nfkb1*
*Cebpb*	4.674	*Atf3, Smad7, Il23a, Ltf, Ddit3, Spib, Il1b, Ccr7, Cxcl1, Cxcl3, Tslp, Nfkb1, Egr1, Zbp1, Nr3c1, Il33*
*Pbx1*	4.529	*Smad7, Egr1, Ccr7, Atf3, Nr3c1, Mafb, Cxcl1, Nfkb1, Il33, Ccr2, Ltf, Ifng, Irg1, Tslp, Parp12, Tlr2, Zbp1, Tlr3, Cxcl3*
*Yy1*	4.118	*Egr1, Nr3c1, Tlr2, Mafb, Smad7, Ccr2, Il23a, Atf3, Zbp1*
*Cebpa*	4.080	*Il23a, Ddit3, Atf3, Ccr2, Il1b*
*Tbx5*	4.042	*Cxcr3, Ddit3, Nfkb1, Irf7, Ccr7, Nr3c1, Smad7, Plscr1, Atf3*
*Scrt2*	3.999	*Nfkb1, Ccr7, Nr3c1, Irf7*

### The TLR2/IRF7 Signaling Axis Potentially Mediates Neuroinflammation After SAH

**FIGURE 5:**
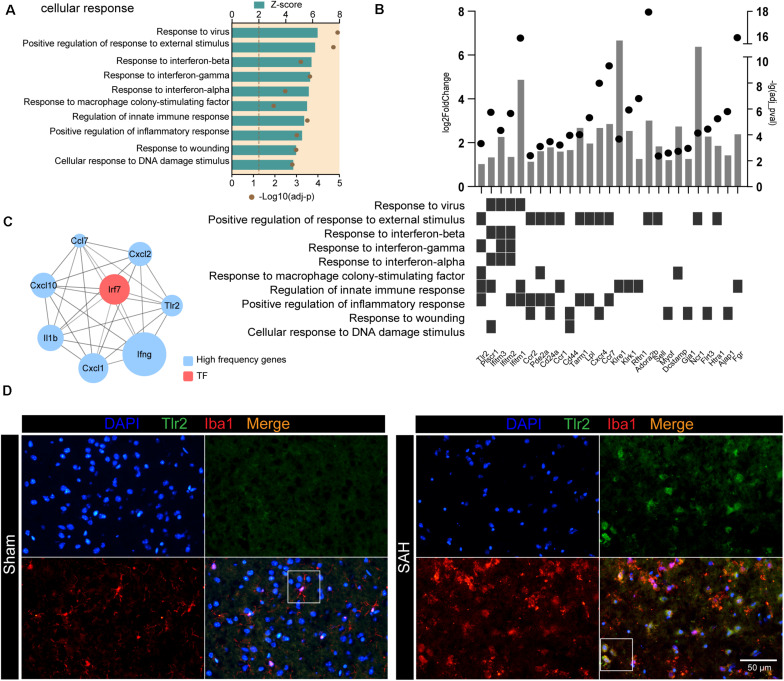
TLR2/IRF7 signaling axis potentially mediates neuroinflammation after SAH. **(A)** GO enrichment analysis was performed by Metascape on all DEGs in sham and SAH microglia. Shown are the *z*-scores of biological functions on cell response-related pathways. **(B)** Expression profiles of DEGs (fold change > 2, adj_pval < 0.01) in SAH microglia related to cell response-related pathways. **(C)** Top 10 hub genes identified by CytoHubba. **(D)** Immunofluorescence staining results of Tlr2 and Iba1 in sham and SAH mice, *n* = 3 per mice group. DEG, differentially expressed gene.

Following SAH, microglia were activated to cope with the disruption of brain homeostasis. We examined the GO terms related to “cellular response” and found that response to stimulus (e.g., response to virus and response to external stimulus) and response to cytokines (e.g., response to interferon and response to macrophage colony-stimulating factor) were enriched in post-SAH microglia (Figure 5A). We also examined SAH-induced gene expression changes of receptor on the membrane in microglia (Figure 5B), most of which were upregulated (e.g., *Bst1*, *Gpnmb*, *Fgr*, and *Tlr2*). Figure 5B also showed that several receptors participated in microglia “cellular response,” including response to external stimulus and response to interferon. Additionally, hub genes of the selected high-frequency genes were identified by CytoHubba, and the top 10 genes were calculated based on Degree algorithm (Figure 5C and Supplementary Table 4). All of the hub genes were upregulated except *Tlr3*, and *Irf7* was the only one identified as TF. In addition, *Tlr2* was also regarded as a hub gene, and the interaction between TLR2 and IRF7 indicates that the TLR2/IRF7 signaling axis may mediate the immune inflammatory response and chemotaxis in post-SAH microglia. Furthermore, immunostaining confirmed that the expression of TLR2 was elevated and colocalized with Iba1 in post-SAH brain (Figure 5D).

## Discussion

In this work, we presented, for the first time, genome-wide transcriptional analyses of microglia isolated from the brain at 72 h after SAH, compared with sham mice. The results demonstrated that (i) post-SAH microglia have robust transcriptomic changes that drive them into an activated state; (ii) transcriptional alteration contributes to SAH-induced neuroinflammation, especially the functional terms related to immune inflammatory response and chemotaxis; and (iii) the TLR2/IRF7 signaling axis may mediate the microglial activation.

Previous studies have reported that microglia contribute to both neuroinflammation and brain recovery, during the acute stage and recovery phase after stroke, respectively (Jiang et al., 2020; Xu et al., 2020). Elmore et al. (2014) found that microglia depletion by PLX3397 for either 21 days or 2 months does not affect learning, memory, motor function, or behavior in adult mice. Furthermore, the blood–brain barrier (BBB) remains intact in microglia-depleted mice (Elmore et al., 2014). However, other researchers claimed that microglia depletion could have unexpected effects, including increased pro- and anti-inflammatory cytokines, circadian system disruption, and increased BBB leakage under hypoxia condition (Miron and Priller, 2020; Sominsky et al., 2021; Yegla et al., 2021). In our study, pharmacological depletion of microglia using PLX3397 ameliorates short-term neurological deficits in post-SAH mice, and long-term neurological functions still need to be tested.

Since microglia are heterogeneous cells, many specific markers are pathologically decreased. However, the expression level of CD45 is increased in injury conditions (Plemel et al., 2020). Therefore, we choose CD45^+^CD11b^+^ to sort out microglia in this study.

We discovered dramatic transcriptomic changes between microglia in the post-SAH and sham mice. A total of 1576 DEGs were identified (6.85% of total ∼23,000 genes), including 928 upregulated DEGs and 648 downregulated DEGs. The functional enrichment analysis showed that biological processes related to inflammatory response were significantly enriched, including inflammatory response, regulation of defense response, chemotaxis, leukocyte migration, cytokine production, and chemokine production. Flow cytometry data examined the infiltration of neutrophils and monocytes into the cortex at 72 h after SAH. These results suggested that microglia switched to pro-inflammatory state at 72 h after SAH compared to sham mice. As expected, this finding is consistent with previous studies that showed increased pro-inflammatory cytokines after SAH (Zheng et al., 2020). However, the underlying mechanisms of these processes remain elusive.

IRF7, as a TF, mainly plays a role in interferon production pathway. IRF7 is also involved in apoptosis and TLR4 pathway. According to the analysis conducted by IRegulon, IRF7 was predicted to be a master regulator whose targets are involved in immune response and cytokine production (e.g., *Cd40*, *Il1b*, *Cxcl10*, *Ifng*, *Ccl6*, *Il33*, and *Tlr2*). Besides, *Irf7* was also predicted to be a hub gene mediating inflammatory response and chemokines. We examined the protein expression level of IRF7 in post-SAH microglia. Some of these effects have been previously reported. Sin et al. (2020) reported that IRF7 promotes IL-1β production. CXCL10 production is IRF7-dependent in macrophages (Tsiantoulas et al., 2018). IRF7 also participates in monocyte differentiation and other inflammatory cytokine production (e.g., TNF-α, IL6, CCL2, and IL33) (Ning et al., 2011; Sun et al., 2014; Simons et al., 2019). Tanaka et al. (2015) demonstrated that the expression level of IRF7 increased during the M2 to M1-like switch in microglia. However, another study found that Irf7 expression induced by spinal cord injury reduced microglial pro-inflammatory activity (Cohen et al., 2014).

Toll-like receptor 2 (TLR2), a member of the TLR family, recognizes PAMPs and DAMPs, leading to upregulation of signaling pathways to modulate inflammatory response (Lalancette-Hebert et al., 2017). We observed an increasing expression level of *Tlr2* in microglia at 72 h after SAH, and it participated in biological processes, such as the cellular response to interferon, response to external stimulus, defense response, inflammatory response, cytokine production, and chemotaxis. According to the results from CytoHubba, *Tlr2* was confirmed as a hub gene in activating immune inflammatory response and pathways related to cytokines and chemokines. Consistent with the results of the present study, Jiang et al. (2020) have reported an elevated expression level of *Tlr2* in microglia purified from post-ischemic stroke mice. Mottahedin et al. (2019) and Deng et al. (2020) have demonstrated that TLR2 participates in microglia activation and peripheral immune cell infiltration in ischemic stroke. Administration of the inhibitor targeting TLR2 decreased the release of pro-inflammatory cytokines (Wang et al., 2020).

According to the results from the PPI network and CytoHubba calculation, we identified the TLR2/IRF7 signaling axis that potentially mediates an inflammatory response in microglia after SAH. As previous studies reported, TLR2 activation induces IFN *via* IRF7, leading to CXCL10 production (Dietrich et al., 2010).

Several limitations of our study should be noted. Firstly, bulk RNA-seq was performed on the sorted cells, which measured the average expression level in a sample, thus limiting us to distinguish the subpopulations of cells within each sample. Combining bulk RNA-seq with single-cell RNA-seq technology may help to improve this problem. Secondly, because microglia are heterogeneous, it is hard to distinguish them from infiltration macrophages and perivascular macrophages, and more specific markers should be considered to refine our FACS strategy. Thirdly, we obtained the data from a single time point (3 days) after SAH, because the number of microglia reached the maximum at 72 h (Xu et al., 2019). However, 24 h after SAH is a critical time point to investigate microglial activation, and further studies should expand the time window to confirm the protein expression levels and investigate microglia transcriptional profiles at different time points. Fourthly, the verification of the TLR2/IRF7 signaling axis is limited; *in vivo* and *in vitro* functional/mechanical experiments should be conducted in further studies.

In summary, we report that microglia at 72 h after SAH harbor robust transcriptional changes compared to sham mice. The alteration in post-SAH microglia genes may contribute to immune inflammatory response, cytokine and chemokine production, and chemotaxis, which then lead to a poor outcome. The TLR2/IRF7 signaling axis is considered to be capable of regulating neuroinflammatory processes after SAH. Based on these findings, further investigation targeting the TLR2/IRF7 axis may help to improve the outcome of SAH patients.

## Data Availability Statement

The datasets generated for this study can be found in online repositories. The names of the repository/repositories and accession number(s) can be found below: https://www.ncbi.nlm.nih.gov/geo/query/acc.cgi?acc=GSE167957.

## Ethics Statement

The animal study was reviewed and approved by Institutional Ethics Committee of the Second Affiliated Hospital, Zhejiang University School of Medicine.

## Author Contributions

SX, SM, and JL drafted the manuscript. HW and XD reviewed and modified the manuscript. LS, JYZ, and JMZ revised the manuscript. All authors agreed on the final version.

## Conflict of Interest

The authors declare that the research was conducted in the absence of any commercial or financial relationships that could be construed as a potential conflict of interest.

## Footnotes

1 http://genome.ucsc.edu/

2 http://metascape.org/

3 http://string-db.org/

## References

[B1] Agalave N. M. Lane B. T. Mody P. H. Szabo-Pardi T. A. Burton M. D. (2020). Isolation, culture, and downstream characterization of primary microglia and astrocytes from adult rodent brain and spinal cord. *J. Neurosci. Methods* 340:108742. 10.1016/j.jneumeth.2020.108742 32315669 PMC7293863

[B2] Amodio S. Bouzat P. Robba C. Taccone F. S. (2020). Rethinking brain injury after subarachnoid hemorrhage. *Crit. Care* 24:612. 10.1186/s13054-020-03342-2 PMC7568360 33069252

[B3] Barbalat R. Lau L. Locksley R. M. Barton G. M. (2009). Toll-like receptor 2 on inflammatory monocytes induces type I interferon in response to viral but not bacterial ligands. *Nat. Immunol.* 10 1200–1207. 10.1038/ni.1792 19801985 PMC2821672

[B4] Bhattacharya S. Dunn P. Thomas C. G. Smith B. Schaefer H. Chen J. (2018). ImmPort, toward repurposing of open access immunological assay data for translational and clinical research. *Sci. Data* 5:180015. 10.1038/sdata.2018.15 PMC5827693 29485622

[B5] Bouet V. Boulouard M. Toutain J. Divoux D. Bernaudin M. Schumann-Bard P. (2009). The adhesive removal test: a sensitive method to assess sensorimotor deficits in mice. *Nat. Protoc.* 4 1560–1564. 10.1038/nprot.2009.125 19798088

[B6] Chang C. Z. Wu S. C. Kwan A. L. (2014). Glycyrrhizin attenuates toll like receptor-2, -4 and experimental vasospasm in a rat model. *J. Immunol. Res.* 2014:740549. 10.1155/2014/740549 PMC4134788 25152897

[B7] Chang C. Z. Wu S. C. Kwan A. L. (2015). A purine antimetabolite attenuates toll-like receptor-2, -4, and subarachnoid hemorrhage-induced brain apoptosis. *J. Surg. Res.* 199 676–687. 10.1016/j.jss.2015.06.011 26163325

[B8] Cohen M. Matcovitch O. David E. Barnett-Itzhaki Z. Keren-Shaul H. Blecher-Gonen R. (2014). Chronic exposure to TGFbeta1 regulates myeloid cell inflammatory response in an IRF7-dependent manner. *EMBO J.* 33 2906–2921. 10.15252/embj.201489293 25385836 PMC4282639

[B9] Deng W. Mandeville E. Terasaki Y. Li W. Holder J. Chuang A. T. (2020). Transcriptomic characterization of microglia activation in a rat model of ischemic stroke. *J. Cereb. Blood Flow Metab.* 40 S34–S48. 33208001 10.1177/0271678X20932870 PMC7687036

[B10] Dietrich N. Lienenklaus S. Weiss S. Gekara N. O. (2010). Murine toll-like receptor 2 activation induces type I interferon responses from endolysosomal compartments. *PLoS One* 5:e10250. 10.1371/journal.pone.0010250 20422028 PMC2857745

[B11] Elmore M. R. Najafi A. R. Koike M. A. Dagher N. N. Spangenberg E. E. Rice R. A. (2014). Colony-stimulating factor 1 receptor signaling is necessary for microglia viability, unmasking a microglia progenitor cell in the adult brain. *Neuron* 82 380–397. 10.1016/j.neuron.2014.02.040 24742461 PMC4161285

[B12] Fujii M. Yan J. Rolland W. B. Soejima Y. Caner B. Zhang J. H. (2013). Early brain injury, an evolving frontier in subarachnoid hemorrhage research. *Transl. Stroke Res.* 4 432–446. 10.1007/s12975-013-0257-2 23894255 PMC3719879

[B13] Janky R. Verfaillie A. Imrichova H. Van de Sande B. Standaert L. Christiaens V. (2014). iRegulon: from a gene list to a gene regulatory network using large motif and track collections. *PLoS Comput. Biol.* 10:e1003731. 10.1371/journal.pcbi.1003731 25058159 PMC4109854

[B14] Jiang L. Mu H. Xu F. Xie D. Su W. Xu J. (2020). Transcriptomic and functional studies reveal undermined chemotactic and angiostimulatory properties of aged microglia during stroke recovery. *J. Cereb. Blood Flow Metab.* 40 S81–S97. 32065074 10.1177/0271678X20902542 PMC7687033

[B15] Lalancette-Hebert M. Faustino J. Thammisetty S. S. Chip S. Vexler Z. S. Kriz J. (2017). Live imaging of the innate immune response in neonates reveals differential TLR2 dependent activation patterns in sterile inflammation and infection. *Brain Behav. Immun.* 65 312–327. 10.1016/j.bbi.2017.05.020 28579520 PMC6151183

[B16] Leek J. T. Johnson W. E. Parker H. S. Jaffe A. E. Storey J. D. (2012). The sva package for removing batch effects and other unwanted variation in high-throughput experiments. *Bioinformatics* 28 882–883. 10.1093/bioinformatics/bts034 22257669 PMC3307112

[B17] Liu W. Li R. Yin J. Guo S. Chen Y. Fan H. (2019). Mesenchymal stem cells alleviate the early brain injury of subarachnoid hemorrhage partly by suppression of Notch1-dependent neuroinflammation: involvement of Botch. *J. Neuroinflamm.* 16:8. 10.1186/s12974-019-1396-5 PMC6334441 30646897

[B18] Love M. I. Huber W. Anders S. (2014). Moderated estimation of fold change and dispersion for RNA-seq data with DESeq2. *Genome Biol.* 15:550. 10.1186/s13059-014-0550-8 PMC4302049 25516281

[B19] Lu J. Sun Z. Fang Y. Zheng J. Xu S. Xu W. (2019). Melatonin suppresses microglial necroptosis by regulating deubiquitinating enzyme A20 after intracerebral hemorrhage. *Front. Immunol.* 10:1360. 10.3389/fimmu.2019.01360 31258534 PMC6587666

[B20] Macdonald R. L. Schweizer T. A. (2017). Spontaneous subarachnoid haemorrhage. *Lancet* 389 655–666. 27637674 10.1016/S0140-6736(16)30668-7

[B21] Miron V. E. Priller J. (2020). Investigating microglia in health and disease: challenges and opportunities. *Trends Immunol.* 41 785–793. 10.1016/j.it.2020.07.002 32736967

[B22] Mottahedin A. Joakim E. K. C. Truve K. Hagberg H. Mallard C. (2019). Choroid plexus transcriptome and ultrastructure analysis reveals a TLR2-specific chemotaxis signature and cytoskeleton remodeling in leukocyte trafficking. *Brain Behav. Immun.* 79 216–227. 10.1016/j.bbi.2019.02.004 30822467 PMC6591031

[B23] Muroi C. Fujioka M. Marbacher S. Fandino J. Keller E. Iwasaki K. (2015). Mouse model of subarachnoid hemorrhage: technical note on the filament perforation model. *Acta Neurochir. Suppl.* 120 315–320. 10.1007/978-3-319-04981-6_54 25366644

[B24] Najafi A. R. Crapser J. Jiang S. Ng W. Mortazavi A. West B. L. (2018). A limited capacity for microglial repopulation in the adult brain. *Glia* 66 2385–2396. 10.1002/glia.23477 30370589 PMC6269202

[B25] Ning S. Pagano J. S. Barber G. N. (2011). IRF7: activation, regulation, modification and function. *Genes Immun.* 12 399–414. 10.1038/gene.2011.21 21490621 PMC4437765

[B26] Plemel J. R. Stratton J. A. Michaels N. J. Rawji K. S. Zhang E. Sinha S. (2020). Microglia response following acute demyelination is heterogeneous and limits infiltrating macrophage dispersion. *Sci. Adv.* 6:eaay6324. 10.1126/sciadv.aay6324 31998844 PMC6962036

[B27] Rass V. Helbok R. (2019). Early brain injury after poor-grade subarachnoid hemorrhage. *Curr. Neurol. Neurosci. Rep.* 19:78. 10.1007/s11910-019-0990-3 PMC6715808 31468197

[B28] Rayasam A. Faustino J. Lecuyer M. Vexler Z. S. (2020). Neonatal stroke and TLR1/2 ligand recruit myeloid cells through the choroid plexus in a CX3CR1-CCR2- and context-specific manner. *J. Neurosci.* 40 3849–3861. 10.1523/jneurosci.2149-19.2020 32269105 PMC7204080

[B29] Schallner N. Pandit R. LeBlanc R. III Thomas A. J. Ogilvy C. S. Zuckerbraun B. S. (2015). Microglia regulate blood clearance in subarachnoid hemorrhage by heme oxygenase-1. *J. Clin. Invest.* 125 2609–2625. 10.1172/jci78443 26011640 PMC4563677

[B30] Schneider U. C. Davids A. M. Brandenburg S. Muller A. Elke A. Magrini S. (2015). Microglia inflict delayed brain injury after subarachnoid hemorrhage. *Acta Neuropathol.* 130 215–231. 10.1007/s00401-015-1440-1 25956409

[B31] Shannon P. Markiel A. Ozier O. Baliga N. S. Wang J. T. Ramage D. (2003). Cytoscape: a software environment for integrated models of biomolecular interaction networks. *Genome Res.* 13 2498–2504. 10.1101/gr.1239303 14597658 PMC403769

[B32] Shi L. Liang F. Zheng J. Zhou K. Chen S. Yu J. (2018). Melatonin regulates apoptosis and autophagy via ROS-MST1 pathway in subarachnoid hemorrhage. *Front. Mol. Neurosci.* 11:93. 10.3389/fnmol.2018.00093 29632474 PMC5879134

[B33] Simons K. H. de Vries M. R. de Jong R. C. M. Peters H. A. B. Jukema J. W. Quax P. H. A. (2019). IRF3 and IRF7 mediate neovascularization via inflammatory cytokines. *J. Cell Mol. Med.* 23 3888–3896. 10.1111/jcmm.14247 30932349 PMC6533520

[B34] Sin W. X. Yeong J. P. Lim T. J. F. Su I. H. Connolly J. E. Chin K. C. (2020). IRF-7 mediates Type I IFN responses in Endotoxin-challenged mice. *Front. Immunol.* 11:640. 10.3389/fimmu.2020.00640 32373120 PMC7176903

[B35] Sokol B. Wasik N. Jankowski R. Holysz M. Wieckowska B. Jagodzinski P. (2016). Soluble Toll-Like receptors 2 and 4 in cerebrospinal fluid of patients with acute hydrocephalus following aneurysmal subarachnoid haemorrhage. *PLoS One* 11:e0156171. 10.1371/journal.pone.0156171 27223696 PMC4880192

[B36] Sominsky L. Dangel T. Malik S. De Luca S. N. Singewald N. Spencer S. J. (2021). Microglial ablation in rats disrupts the circadian system. *FASEB J.* 35:e21195. 10.1096/fj.202001555RR 33200466

[B37] Soulet D. Rivest S. (2008). Microglia. *Curr. Biol.* 18 R506–R508. 18579087 10.1016/j.cub.2008.04.047

[B38] Sugawara T. Ayer R. Jadhav V. Zhang J. H. (2008). A new grading system evaluating bleeding scale in filament perforation subarachnoid hemorrhage rat model. *J. Neurosci. Methods* 167 327–334. 10.1016/j.jneumeth.2007.08.004 17870179 PMC2259391

[B39] Sun L. Zhu Z. Cheng N. Yan Q. Ye R. D. (2014). Serum amyloid A induces interleukin-33 expression through an IRF7-dependent pathway. *Eur. J. Immunol.* 44 2153–2164. 10.1002/eji.201344310 24777946 PMC4118754

[B40] Szklarczyk D. Franceschini A. Wyder S. Forslund K. Heller D. Huerta-Cepas J. (2015). STRING v10: protein-protein interaction networks, integrated over the tree of life. *Nucleic Acids Res.* 43 D447–D452. 25352553 10.1093/nar/gku1003 PMC4383874

[B41] Tanaka T. Murakami K. Bando Y. Yoshida S. (2015). Interferon regulatory factor 7 participates in the M1-like microglial polarization switch. *Glia* 63 595–610. 10.1002/glia.22770 25422089

[B42] Tsiantoulas D. Sage A. P. Goderle L. Ozsvar-Kozma M. Murphy D. Porsch F. (2018). B Cell-activating factor neutralization aggravates atherosclerosis. *Circulation* 138 2263–2273. 10.1161/circulationaha.117.032790 29858401 PMC6181204

[B43] Wang X. Tian S. Wang H. Liu P. Zheng H. Wu L. (2020). Botulinum toxin type A alleviates neuropathic pain and suppresses inflammatory cytokines release from microglia by targeting TLR2/MyD88 and SNAP23. *Cell Biosci.* 10:141. 10.1186/s13578-020-00501-4 PMC7724852 33298171

[B44] Xu W. Mo J. Ocak U. Travis Z. D. Enkhjargal B. Zhang T. (2020). Activation of Melanocortin 1 receptor attenuates early brain injury in a rat model of subarachnoid hemorrhage viathe suppression of neuroinflammation through AMPK/TBK1/NF-kappaB pathway in rats. *Neurotherapeutics* 17 294–308. 10.1007/s13311-019-00772-x 31486022 PMC7007470

[B45] Xu Z. Shi W. H. Xu L. B. Shao M. F. Chen Z. P. Zhu G. C. (2019). Resident Microglia activate before peripheral Monocyte infiltration and p75NTR blockade reduces microglial activation and early brain injury after subarachnoid hemorrhage. *ACS Chem. Neurosci.* 10 412–423. 10.1021/acschemneuro.8b00298 30117729

[B46] Yegla B. Boles J. Kumar A. Foster T. C. (2021). Partial microglial depletion is associated with impaired hippocampal synaptic and cognitive function in young and aged rats. *Glia* 69 1494–1514. 10.1002/glia.23975 33586813 PMC8278544

[B47] Zheng Z. V. Lyu H. Lam S. Y. E. Lam P. K. Poon W. S. Wong G. K. C. (2020). The dynamics of Microglial polarization reveal the resident neuroinflammatory responses after subarachnoid hemorrhage. *Transl. Stroke Res.* 11 433–449. 10.1007/s12975-019-00728-5 31628642

